# Healthy Weight Loss for the Cancer Survivor

**Published:** 2014-07-01

**Authors:** Kristy K. Hager

**Affiliations:** Ms. Hager is an oncology dietician at Wellmont Cancer Institute in Kingsport, Tennessee

For most oncology patients, weight gain is not considered problematic. However, in some instances weight gain can be detrimental to health. Outpatient oncology dietitians most often work with patients who are currently undergoing treatment. Weight loss during cancer treatment is associated with incomplete treatments, increased toxicity from treatments, and decreased rates of survival (Marian, 2013). But once patients transition into survivorship, weight loss is often needed.

Obesity contributes to many health issues. It has been associated with an increased risk for recurrence of colorectal and breast cancers (in postmenopausal women; Marian, 2013). Obesity "increases the risk of cancer death among postmenopausal women with estrogen receptor–positive (ER+) breast cancer" (Fuentes-Mattei et al., 2014). It has also been "associated with significantly increased risk of progression beyond the confirmatory biopsy" for prostate cancer (Bhindi et al., 2014). As health-care practitioners, we want our patients to be as healthy as possible and to have the least chance for recurrence of their disease.

The American Institute for Cancer Research (AICR) and the Academy of Nutrition and Dietetics (AND, formerly the American Dietetic Association) provide guidelines for cancer survivors and recommendations to reduce cancer risk. A synopsis of these recommendations can be found in Table 1 .

**Table 1:**
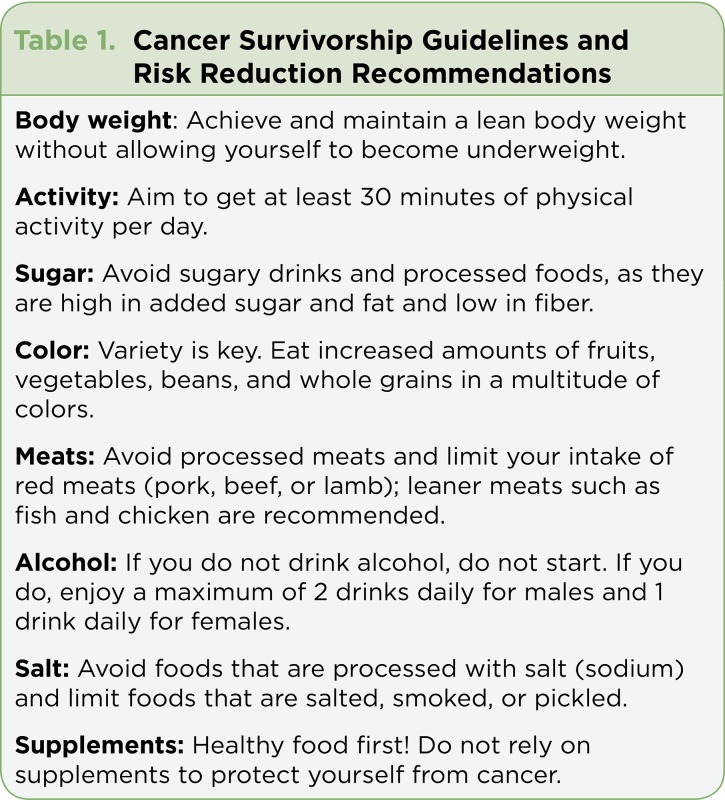
Cancer Survivorship Guidelines and Risk Reduction Recommendations

## Avoid ’Fad’ Diets

Do a quick Google search for diets, and you will be barraged with "cure-alls." You might find the South Beach Diet, the Grapefruit Diet, the Cabbage Diet, and even the No Diet approach. In our society, instant gratification is not fast enough. But successful weight loss requires at least two components: It must be gradual, and it requires work. If a diet or product sounds too good to be true, it usually is (AND, 2012).

But there are many weight loss options available in today’s society. How do you know what options are safe? How can you be sure you are recommending the right path for your patients? This article will provide some tips to help navigate safe and effective weight loss for the cancer survivor.

One of the most popular diets as of late is the Human Chorionic Gonadotropin (HCG) Diet. According to an article in the May 2013 issue of the Annals of Pharmacotherapy, HCG gained popularity in the 1950s. Dr. Albert Simeons promoted injections in combination with an ultra-low-calorie diet consisting of 500 calories per day (Goodbar, Foushee, Eagerton, Haynes, & Johnson, 2013). According to the article, "this weight loss strategy claims to redistribute body fat from the hips, thighs, and stomach, without undesirable effects such as hunger and irritability" (Goodbar et al., 2013). But the public needs to beware of the dangers associated with the use of higher amounts of HCG (Goodbar et al., 2013). In fact, in the same article mentioned above, there is a report of an incidence of deep vein thrombosis and bilateral pulmonary embolism associated with the initiation of the HCG diet (Goodbar et al., 2013). This and other similar products are increasingly available to the public and importantly, are not regulated by the US Food and Drug Administration (FDA). This means that there are no published safety or efficacy data among patients using it as a weight loss aid.

## Be Wary of ’Harmless’ Supplements

Just because a supplement says it is "all-natural," it should not be considered harmless. Some ingredients in herbal supplements may be harmful in specific situations. For example, supplements containing hoodia, a cactus found in southeastern Africa, are being promoted for weight loss. However, a compound in hoodia can inhibit the enzyme CYP3A4 and in turn affect the concentration of drugs metabolized by this enzyme (Memorial Sloan Kettering Cancer Center [MSKCC], 2014).

Another supplement that should be avoided is ephedra, which has long been used as a medicinal herb in China and India. It has anti-inflammatory and antibacterial properties and has been promoted as a natural stimulant and appetite suppressant (MSKCC, 2014). But overdose and misuse of ephedra has resulted in heart attacks, strokes, seizures, psychosis, and death. In fact, the sale of supplements containing ephedra has been banned by the FDA because of the risk to human health (MSKCC, 2014).

## Maintain a Safe Rate of Weight Loss

Patients cannot expect overnight results. The weight did not appear overnight (although sometimes it feels that way) and will not disappear overnight. There are no magic pills or potions that burn fat while you sleep (AND, 2012). But what is considered a safe rate of weight loss? According to the AND (formerly the American Dietetic Association), a loss of 0.5 to 1 pound per week is healthy. A patient can lose muscle and water with too rapid a weight loss. Plus, rapid weight loss can make a person more likely to regain the pounds quickly after the initial loss (AND, 2012).

## Eat Plant-Based Foods

Eating plant-based foods does not mean one must embrace total vegetarianism. Oncology dietitians recommend plant-based foods because they are rich in nutrients and low in calories. Fruits, vegetables, whole grains, and beans contain fewer calories than sweets. Eating these foods makes it easier to achieve and maintain a healthy weight. Plant-based foods also maximize phytochemicals, which are protective compounds that naturally occur in plants. Patients can start by following the New American Plate standard (AICR, 2013). The New American Plate standard is a portion control guide. A good goal is to consume meals that are 2/3 plant based and 1/3 animal based. The animal-based items should consist of fish, poultry, lean red meats, and cheese. It is recommended to avoid a high intake of processed meats such as cold cuts, bacon, sausage, and ham (AICR, 2013).

## Be Consistent

Patients should not beat themselves up when they have slip-ups, as they run the risk of giving up. Overdoing it one day does not mean all efforts are lost. Patients can be advised to pick up with their healthy eating plan at the very next meal. It is the overall effort that matters. Table 2 provides some general tips for weight loss success.

**Table 2:**
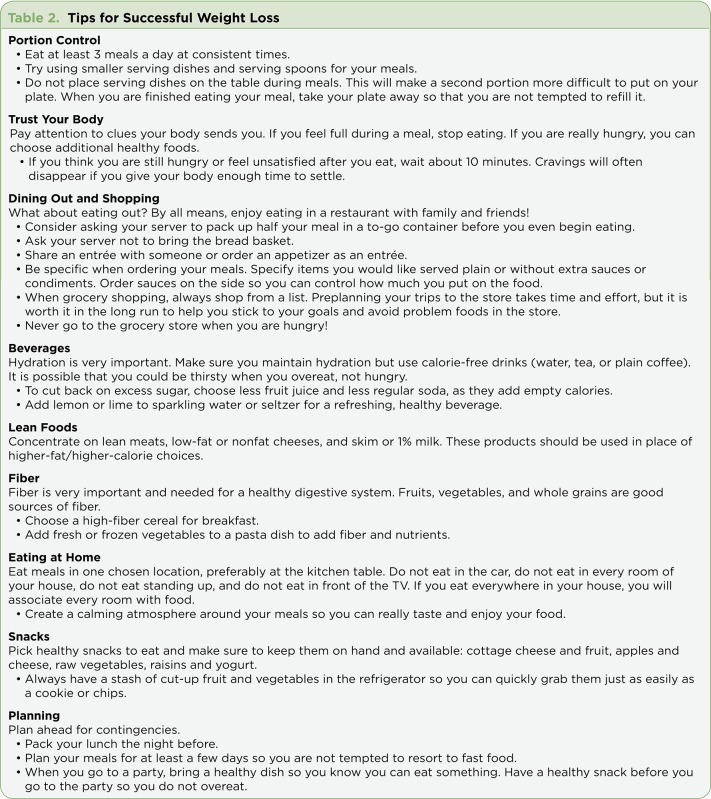
Tips for Successful Weight Loss

## Resources for the Advanced Practitioner

It is believed that diet and exercise behaviors impact outcomes in the cancer survivor (Bhindi et al., 2014; Fuentes-Mattei et al., 2014). However, there is limited evidence-based research on how best to accomplish this frequently elusive goal (McTiernan, 2005). Referral to a registered dietitian who is a certified specialist in oncology (CSO) is ideal and can be beneficial for survivors. To locate a CSO in your area, visit www.oncologynutrition.org. Click on the "Eat Right to Fight Cancer" tab and then "Find an Oncology Dietitian." In the absence of a registered dietitian, good resources are available for the advanced practitioner to guide the cancer survivor toward safe and effective weight loss. These resources are summarized in Table 3.

**Table 3:**
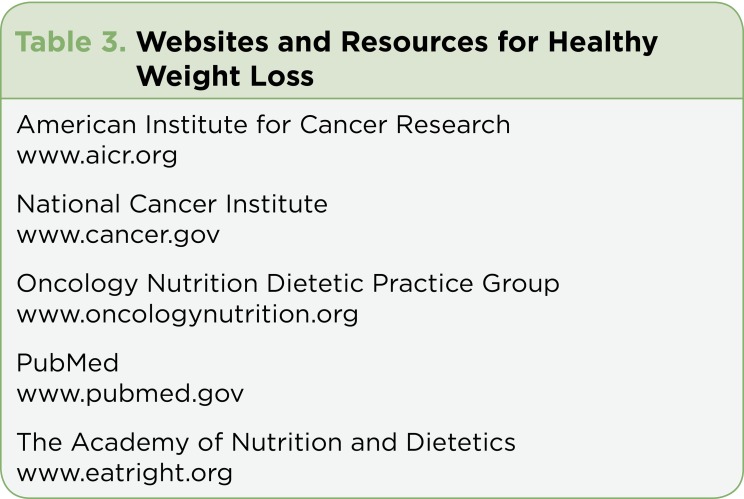
Websites and Resources

## Conclusion

It is well established that maintaining a healthy weight and getting a sufficient amount of exercise are best for cancer survivors and non–cancer survivors alike. But losing weight and perhaps more importantly, keeping it off, can be extremely difficult for many people, especially those who have recently faced the physical and mental challenges of cancer treatment. In addition, different people respond to different motivations and varied weight loss approaches. It is important to guide patients toward healthy, sustainable methods of weight loss that can become lifestyle changes, not fads.
